# Transverse Presentations.—History of a Case with a New Method of Treatment

**Published:** 1892-02

**Authors:** Eugene Everett Barnum

**Affiliations:** Lancaster, C. H., Virginia; Ex-President of the Orleans County (N. Y.) Medical Society; Member of the Central New York Medical Society; Member of the Virginia State Medical Society, etc.


Buffalo Medical and Surgical Journal

Vol. XXXI. FEBRUARY, 1892. No. 7.

### Original Communications.

### TRANSVERSE PRESENTATIONS.—HISTORY OF A CASE WITH A NEW METHOD OF TREATMENT.

By EUGENE EVERETT BARNUM, A. M., M. D.,

Lancaster, C. H., Virginia.

Ex-President of the Orleans County (N. Y.) Medical Society; Member of the Central New York Medical Society; Member of the Virginia State Medical Society, etc.

Of all the cases which a physician is called upon to attend, there are few which give him more trouble, or which cause greater anxiety, than do those of transverse presentations.

Such complications, at all times, are serious; but their gravity is far greater if the patient has been left without medical treatment until convulsions, or threatened collapse, or the unexpected appearance of a hand protruding from the vulva give warning, in terms too plain to be misunderstood, that all is not right, and cause the friends to send a messenger in great haste for a physician.

To the physician who then comes in only—almost—"at the death," a condition is presented, the gravity of which all authorities admit. All agree that delivery by the unaided power of nature is the very rare exception and not the common rule.

Playfair says: "Intervention on the part of the accoucheur is, therefore, absolutely essential, and the safety of both mother and child depends upon the early detection of the abnormal position of the fetus; for the necessary treatment which is comparatively easy and safe before labor has been long in progress, becomes most difficult and hazardous if there has been much delay."

Referring to Churchill's statistics, in speaking of the prognosis and treatment of these cases, Playfair also says:

Shoulder presentations occur about once in 260 cases,—that is, only slightly less frequently than those of the face. The prognosis, both to mother and child, is much more unfavorable, for he estimates that out of

235 cases, one in nine of the mothers and half of the children were lost. The prognosis in each individual case will, of course, vary much with the period of delivery, of which the malposition is recognized. If detected early, interference is easy, and the prognosis ought to be good; whereas, there are few obstetric difficulties more trying than a case of shoulder presentation in which the necessary treatment has been delayed until the presenting part has been tightly joined into the cavity of the pelvis.

In speaking of the mechanism of this presentation, he says: "It is hardly proper to talk of the mechanism of shoulder presentations since, if left unassisted, they almost invariably lead to the gravest consequences."

Dr. Charpentier, in Hood's Cyclopedia of Obstetrics and Gynecology, gives the following under the head of prognosis of this presentation:

The prognosis of delivery by the trunk is always very serious. Besides, we must endeavor in every case to recognize this presentation before labor; for, as we will see, we can transpose it into a vertex presentation by external manipulations. But if labor has commenced and external version is impossible, spontaneous delivery should never be waited for and recourse must be had to version by internal manipulation, which, however, is not always devoid of danger for mother and fetus. Hence, the prognosis is always grave in proportion to the time elapsed since the outset of labor, as the difficulties of intervention increase *pari passu*.

In the article on Labor, in the Reference Hand-book of the Medical Sciences, we find: "The prognosis is not necessarily bad if the treatment be early and judicious. But otherwise the maternal mortality is considerable and the fetal still greater; while in long neglected cases it is well nigh impossible to save the child's life, and the mother's is greatly imperiled . . . When the arm has remained prolapsed for hours, and the uterus has contracted tetanically about it, the waters having entirely drained away, reversion may be impossible. Then, if our examination reveals no tendency toward spontaneous evolution, we have to choose only between Cesarean section and embryotomy."

Other authorities, which I have consulted, give the same testimony. All agree that an attempt should be made to perform reversion, but all admit that the attempt usually fails, unless it is made early in the labor. When this fails, the only methods of treatment recommended are Cesarean section or embryotomy. In the former case the danger to the mother is great, while the latter operation necessarily destroys the child and is far from being without danger to the mother.

I believe that in cases of such gravity any method of treatment is worthy of a trial, if it holds out a prospect of reducing the danger either to the mother or to the child. Therefore, I give the following history of a case which occurred in my practice.

On Thursday, March 27, 1890, I was called to assist in a case of transverse presentation.

From the attending physician, Dr. Thomas D. Eubank, I obtained the following history :

The patient was Margaret Wood, colored, twenty-seven years of age, the mother of three children. Her former pregnancies and labors were normal. This, her fourth, pregnancy was without incident until the middle of her eighth month, when "she fell out of her house-door, face foremost, and felt she had done herself great injury." Her labor began at term, two weeks later. Pains began about 3 P. M. Wednesday, March 26th. The colored midwife was summoned and she came at once. Pains continued all night. Early the next morning the "waters broke and a hand was thrust forward with the pain that broke the waters."

After waiting several hours—the patient meantime having had several convulsions—Dr. Eubank was called. He saw the patient about 3 P. M.,—twenty-four hours after the beginning of labor. Upon examination he found the right shoulder presenting with the hand protruding at the vulva. After vainly trying to perform version he asked for assistance, when I was sent for. I arrived at the bedside of the patient about 7 P. M., and found the arm prolapsed as far as the elbow, the waters drained away, the uterus in a state of tonic contraction, caused (to some extent at least) by the ergot which the midwife had given, and the patient discouraged and nearly exhausted.

We renewed the attempt to perform version and again failed, on account of the firmness with which the shoulder and side of the head were jammed down upon, or just within, the pelvic brim. All attempts to replace the prolapsed arm also failed, on account of the tetanic contraction of the uterus upon its contents.

(In this connection I can state that the force with which the shoulder was crowded into the superior strait, is shown by the fact that several months elapsed before the child could move that arm as freely as its fellow was moved.)

Version having failed, our authorities gave us our choice of two methods of treatment—Cesarean section and embryotomy,—neither of which was practicable in this case. It was believed that the former operation would almost necessarily cause the death of the mother. Instruments for the latter operation were not at hand and could not be procured in time to be of service. In this emergency I determined to try a method which, so far as I know, never has been recommended in the treatment of transverse presentations.

Our failure to perform version was due, as has been stated, to the firmness with which the presenting part was held upon or just within the pelvic brim. How to overcome this difficulty was the problem to be solved. Remembering that my friend, Dr. W. W. Potter, of Buffalo, had met with very gratifying results, in certain cases of gynecological practice, by calling the force of gravity to his aid ; and remembering Dr. Playfair's treatment of prolapse of the cord, by calling the same force to his assistance, I decided to make use of this force.

The woman was placed in the knee-chest position upon the bed, when we again attempted to push back the fetus, hoping either to replace the arm and get a vertex presentation, or else to be able to perform podalic version ; but so firmly was the fetus held in position that we again failed. I next brought the patient on her knees, to the edge of the bed, with a strong assistance at each side. Her head was slowly lowered until her position was nearly inverted—her head almost touching the floor. Then compelling her to breathe rapidly, to prevent her "bearing down" involuntarily during her pains, we soon began to feel the gradual withdrawal of the fetus from the superior strait. Within a very few minutes—probably not more than five—we were able to replace the arm. With the next pain the head presented by spontaneous version. The patient was then directed to "bear down hard," and was placed in bed, the descent of the arm being prevented by means of a finger in the vagina.

Labor proceeded rapidly, and within fifteen or twenty minutes the child was born *alive*.

From a careful study of this case, with the aid of all the obstetric literature that was within my reach, I have formed the following conclusions :

I. This was a typical case of transverse presentation when first seen by my friend, Dr. Eubank, twenty-four hours after the beginning of the labor, and many hours after the hand "was born."

II. In that late stage of the labor, version by the usual methods was a physical impossibility; embryotomy was not possible, on account of the absence of the necessary instruments, and Cesarean section would only hasten the death of the mother.

III. By the method of treatment employed by me the presenting part was removed from the superior strait, the malposition was corrected and the child was born *alive*,—the whole time from the beginning of the treatment to the birth of the child not exceeding thirty minutes.

IV. The proposed method of treatment does not necessitate the exposure of the patient, and if the changes of position are made slowly and carefully, very little, if any, distress or harm will be experienced by her.

V. Special complications may call for special treatment,—for instance, convulsions might require the use of chloroform, etc.

VI. I believe that this method will be found useful in performing version by external manipulation, by internal manipulation or by both combined, and in many cases, as in mine, it will give nature a chance to correct the presentation by spontaneous version.

VII. A failure of this method will not prevent or complicate treatment of Cesarean section or embryotomy.

Whether this method of treating transverse presentations is, as I believe, original with me or not, I can bear testimony to its efficacy in the only case of the kind which ever occurred in my practice.

